# Cerebral nocardiosis with osteomyelitis of skull vault complicating a primary neck lesion in an immunocompetent patient: a case report

**DOI:** 10.11604/pamj.2021.38.349.26635

**Published:** 2021-04-12

**Authors:** Wafa Marrakchi, Abir Aouam, Ikbel Kooli, Hajer Ben Brahim, Adnene Toumi, Mohamed Chakroun

**Affiliations:** 1 Infectious Diseases Department, Fattouma Bourguiba Hospital, Monastir, Tunisia

**Keywords:** Nocardiosis, immunocompetent, osteomyelitis, skull vault, case report

## Abstract

Nocardia infection is an uncommon and rare condition in immunocompetent patient. A case of cutaneous nocardiosis complicated with osteomyelitis of the vault scalp in a 64-year-old man, with no remarkable past medical history, is reported. Treatment with trimethoprime-sulfamethoxazole than doxycycline for 12 months led to complete resolution and no evidence of recurrence was noted. Nocardia infection should be considered even in immunocomptent patients and doxycycline is a good alternative for treatment.

## Introduction

Nocardiosis is an opportunistic, localized or disseminated granulomatous infection caused by an aerobic actinomycete most commonly found in soil, decomposing vegetation and other organic matter, as well as in fresh and salt water [1]. It affects mainly the immunocompromised hosts, including patients with human human immunodeficiency virus (HIV), chronic corticosteroids and persons taking immunosuppressors after transplantation. Primary infection is the most commonly acquired through respiratory tract. Subsequent hematogenous spread may lead to involvement of central nervous system (CNS) and other tissues [2]. The most common sites for dissemination include the CNS (brain), skin and subcutaneous tissues, eyes (especially the retina), kidneys, joints, bones and the heart [3]. Central nervous system involvement is a well-described complication of nocardial infection. However, osteomyelitis is a rare manifestation of *Nocardia* infection. We encountered a very rare case of primary neck cutaneous nocardiosis with soft tissue infection which extended rapidly to the skull and the brain, in an immunocompetent patient.

## Patient and observation

**Figure 1:**
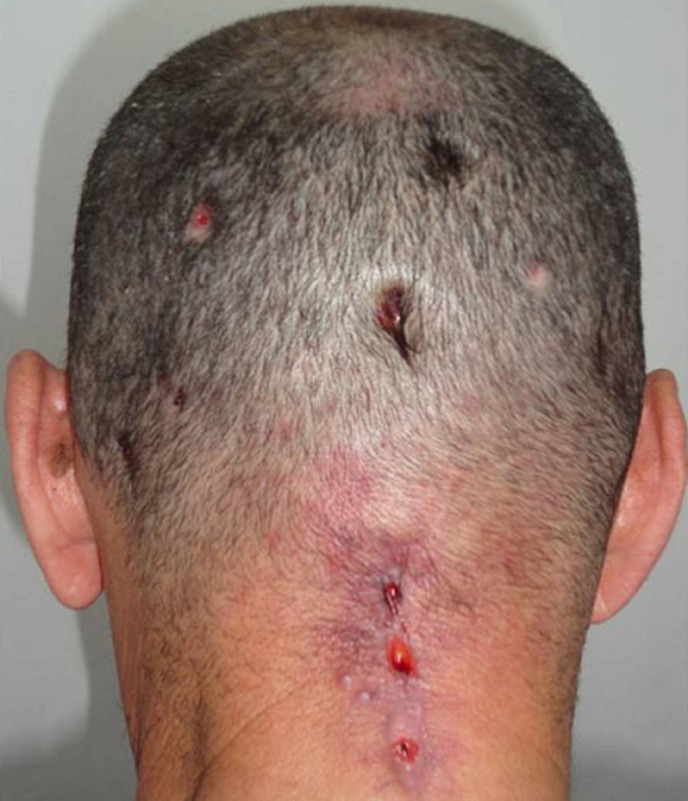
initial presentation showing multiple scalp fistulas

A 64-year-old man with no remarkable past history presented with a raised red lesion over the neck at the site of a prior injury from a tree branch, which had occurred 6 months ago. There was no history of fever or preceding treatment with corticosteroids. He gave history of frequent headaches and pus discharge from the lesion occasionally. He received a course of antibiotics for two months without improvement. The lesion continued to progress and the patient develop scalp fistulas. On physical examination, a solitary 6 x 4 cm subcutaneous well circumscribed lesion in the posterior neck with multiple scalp fistula was seen. There was a pus discharge from the tumefaction and the fistulas and an associated right purulent otorrhoea (Figure 1). Systemic examination was normal. Routine laboratory tests yielded a white blood cells count of 16,600 /mm^3^ and a C-reactive protein of 88.2 mg/l. A computed tomography (CT) scan of the brain showed a hyper-attenuating lesion of the posterior neck which was uniloculated with a liquid component in its centre.

**Figure 2:**
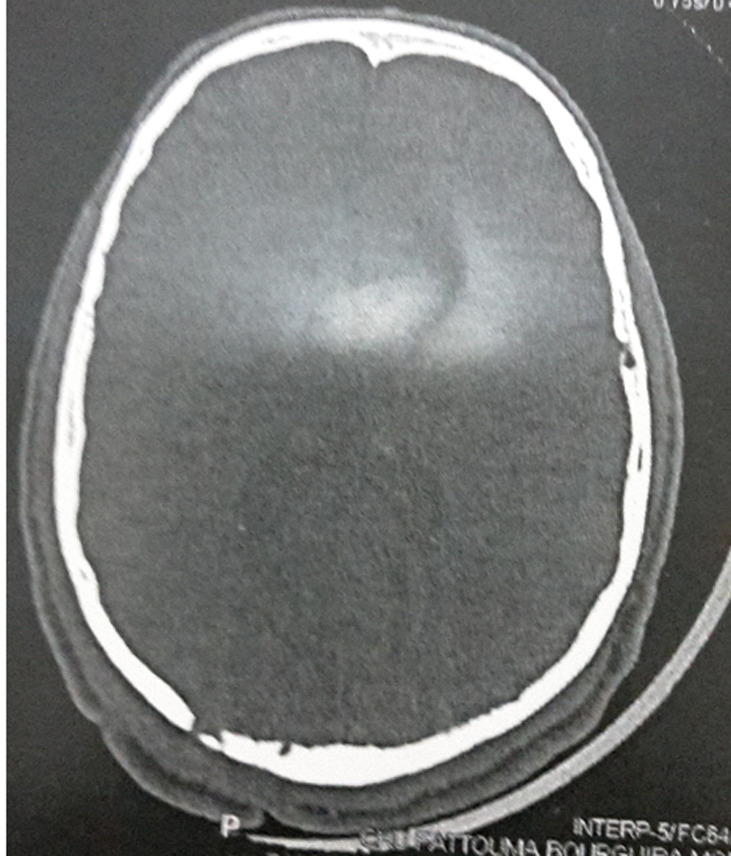
CT-scan osteolysis of the skull in the occipital region near to the primary lesion

**Figure 3:**
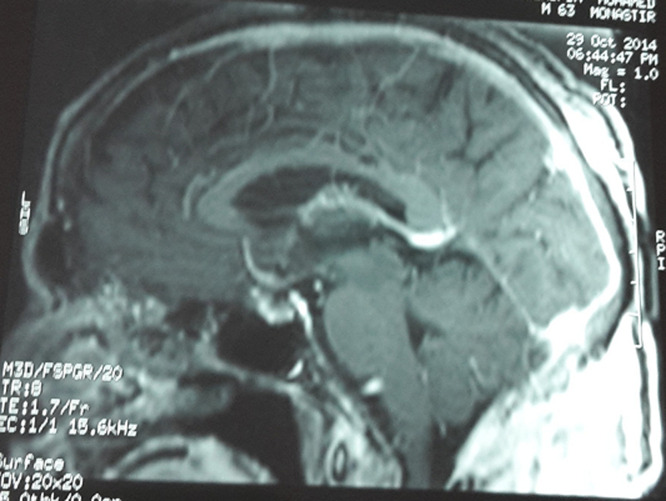
MRI showing superior sagittal sinus thrombosis

There was no evidence of osteolysis of the skull. Magnetic resonance imaging (MRI) examination of the brain and the neck showed a nuchal process that enhanced heterogeneously after IV gadolinium associated with a necrotic component. A clinical diagnosis of actinomycosis, deep mycoses or cutaneous tuberculosis was suggested. But the pyogenic associated infection was suspected because of leukocytosis and the high level of C-reactive protein. He was prescribed a 6 g daily of cefazolin and he underwent skin biopsy which showed a mixed inflammatory infiltrate without necrotizing inflammation or evidence of neoplasia or granuloma. Purified protein derivative (PPD) skin test was negative. Fungal, mycobacterial and bacterial cultures showed no growth. Periodic acid-shiff and acid-fast bacilli stains were negative. Serology for candida, aspergillus and retrovirus were all negative. On follow up, the patient reported headaches, poor concentration and blurring of vision. The lesion continue to progress with pus drainage. Fundoscopy revealed papilloedema. A CT scan of the brain done 15 days later showed an enhancing lesion in the occipital lobe with brain oedema, signs of located meningitis and osteolysis of the skull in the occipital region near to the primary lesion (Figure 2). MRI of brain showed superior sagittal sinus thrombosis (Figure 3). The patient underwent one more skin biopsy and excision of the lesion for bacterial, fungal and mycobacterial culture.

**Figure 4:**
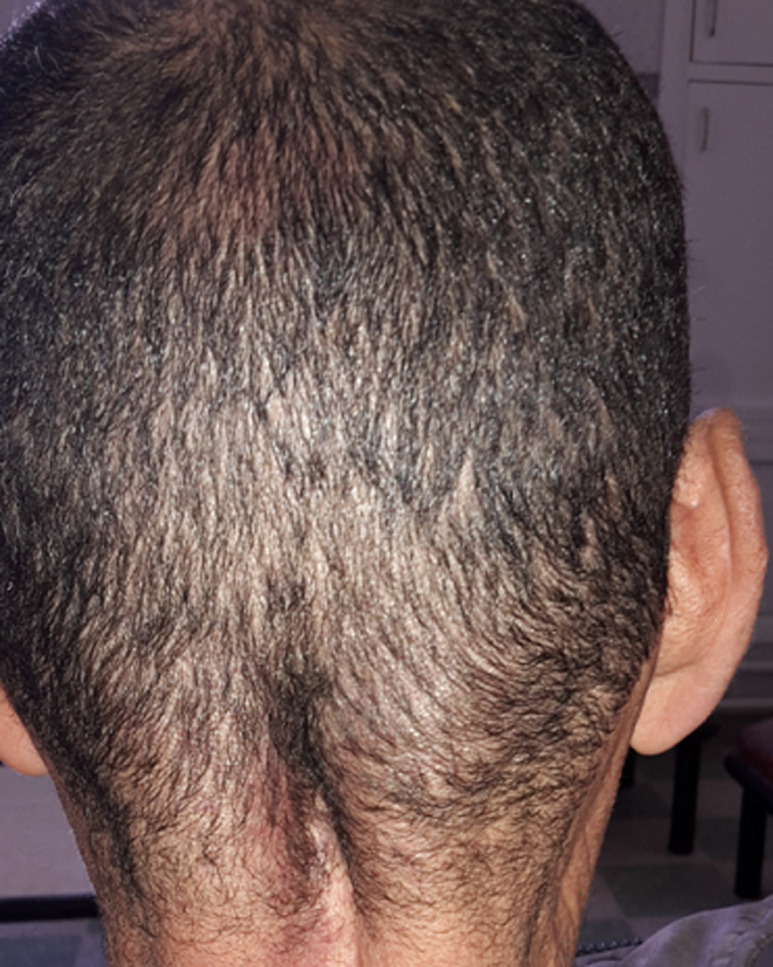
the final outcome after the treatment

On histopathological examination, multiple basophilic filamentous pathogens inside macrophages with dense infiltration by chronic inflammatory cells were seen. These pathogens were periodic acid-shiff positive. A diagnosis of cutaneous nocardiosis was made based on the morphology and the staining properties on biopsy. The patient was started on treatment with sulfamethoxazol SMX (55 mg/kg daily) trimethoprim TMP (10 mg/kg daily) and gentamicin (3 mg/kg daily) for two weeks followed by SMX-TMP for 1 month. The patient was started on anticoagulant therapy followed by oral vitamin K antagonists. He has reported significant improvement and is due for a follow up visit. After nearly four months, the patient presented to the emergency room with high grade fever and an erythematous rash of his trunk and four limbs. A complete blood count revealed a leukocytosis of 19,600/mm^3^ and an elevated absolute eosinophil count of 700/mm^3^. His liver function was deranged with levels of aspartate transaminase 60 UI/ml, alanine transaminase 112 UI/ml and total bilirubin 45 μmol/l. Urine microscopy, renal function tests, blood sugar level, chest X-ray and electrocardiogram were all within normal limits. A peripheral blood smear revealed atypical lymphocytes. Skin biopsy confirmed the diagnosis of drug reaction with eosinphilia systemic symptom syndrome caused by SMX-TMP. The treatment was discontinued and the patient was started with doxycycline 200 mg per day for 18 months. He had shown clinical, biochemical and radiological improvement there were no signs of reactivation of *Nocardia* infection (Figure 4).

## Discussion

Human nocardiosis is generally recognized as an opportunistic disease caused by *Nocardia*, a gram-positive filamentous bacteria. It has usually been associated with immunocompromised states such as underlying malignancy, immunosuppressive therapy, alcoholism, pulmonary disease, diabetes, HIV infection and transplantation recipients [4-6]. The incidence of nocardiosis varies considerably between regions and has been increasing over the past two decades due to the increasing population of immunocompromised individuals [7]. The majority of *Nocardia* infections are caused by inhalation, but some may be acquired by direct inoculation after a contact with soil. Our patient had a history of a prior injury from a tree branch without any predisposing risk factor. More than two-thirds of cases the lungs are the primary site of nocardial infection followed by the skin. Subsequent hematogenous spread may lead to involvement of the central nervous system (CNS) and other tissues. CNS is involved approximately 20 percent of nocardiosis and in 44 percent of disseminated cases [8]. This infection may occur as an isolated CNS lesion without evidence of extracranial disease or as part of a disseminated infection in association with pulmonary or cutaneous diseases [9]. It may present as meningitis, diffuse cerebral infiltration without localized lesions or cerebral abscesses [6]. It generally occur in immunocompromised patients, but several recent reports of this affection in the immunocompetent patients have been reported with more than 50% of patients have no apparent predisposing risk factor [10].

An associated osteomyelitis can be occasionally observed after a direct skin inoculation. It is a rare manifestation and there have been few reported cases of osteomyelitis secondary to nocardiosis [6]. Our patient had presented with a soft tissue infection complicated by meningeal infection and skull vault osteomyelitis. The diagnosis of nocardiosis is readily made by culture of the organism from a biopsy specimen or a lesion aspirate. But, growing of *Nocardia* species in culture is slow and incubation should be carried out for 4-6 weeks. Isolation of this organism and species identification are difficult and require the expertise of a microbiologist. In Tunisia, the nocardiosis is often under diagnosed because stains and cultures are not routinely performed for skin lesion or soft tissue infections. Polymerase chain reactive (PCR) for identification of *Nocardia* can permit faster results than the conventional methods. Most authors recommended early biopsy of lesion to achieve specific identification. Gram positive and acid fast, thin, beaded, branching filaments are the characteristic appearance of *Nocardia* [8]. In our case, the diagnosis was not made by culture of the pathogen from the lesion but it was made by the histopathological examination and direct microscopy. Given the low incidence of *Nocardia* infections, the best therapeutic agent and duration have not been well established in clinical trials. TMP-SMX is the first line option and can therefore be used as an initial empirical treatment in patients with extensive disease, including brain abscess.

Other parenteral drugs with activity against *Nocardia* include amikacin, imipenem, meropenem, ceftriaxone and cefotaxime; each of these drugs can be used as part of combination therapy in severely ill patients. Active oral agents include sulphonamides, minocycline and amoxicillin. Treatment should be guided by antimicrobial sensitivity testing and aggressive attempts to grow the organism should therefore be made. So, TMP-SMX is the drug of the choice; alternative maintenance regimens have not been systematically evaluated, although doxycyline 200 mg daily is a possible alternative. In our case, combined therapy was conducted due to the late diagnosis. Than due to the allergic reaction caused by TMP-SMX, the patient was being treated with doxycycline. The optimal duration of antimicrobial treatment for severe disease has not been early settled. Drugs should be switched to oral medication 3 to 6 weeks after initial endovenous therapy and maintained for at least 6 to 12 months in the cases of cerebral or extensive disease. Our patient was treated for 18 months with a favourable outcome and no relapse was noted.

## Conclusion

Nocardiosis is an uncommon disease and diagnosis requires high levels of suspicion and experience of laboratory staff. Delay to establishing the correct diagnosis occurs frequently due to the non-specific and diverse clinical presentation of nocardiosis and the inherent difficulty in cultivating *Nocardia*. Early biopsy must be indicated to achieve definitive identification and ensure positive outcome.
